# Supplementary material for: A thermostable Cas9 with increased lifetime in human plasma
Source: Nat Commun. 2017 Nov 10;8:1424. doi: 10.1038/s41467-017-01408-4 (PMC5681539; doi:10.1038/s41467-017-01408-4)
Supplement: Supplementary file 1 — Supplementary Information [file 41467_2017_1408_MOESM1_ESM.pdf]

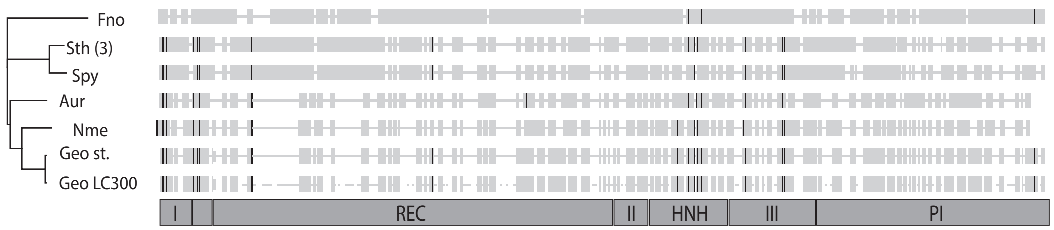

Supplementary Figure 1 | Alignment of selected Cas9 homologs.

Alignment was generated using MAFFT and the approximate domain boundies are shown below. Black lines indicate conserved residues.

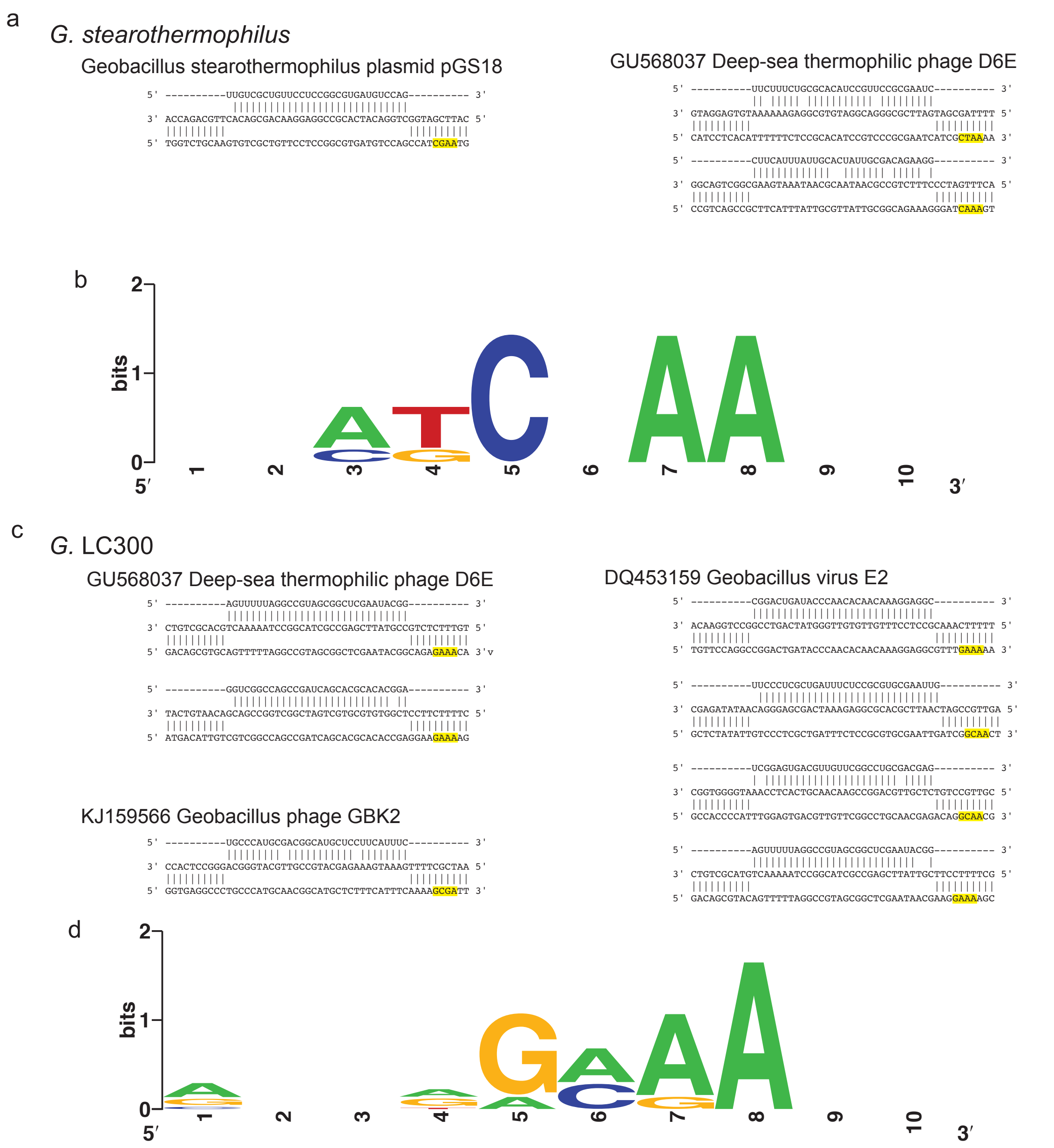

Supplementary Figure 2 | Targets used to generate logos in figure 2b and 2c. a,c. Phage and plasmid targets matching the *G. stearothermophilus* and *G.* LC300 spacer sequences. PAM region is highlighted in yellow. **b,d,** Logo of the sequences 3′ of the protospacer targets identified in **a** and **c.**

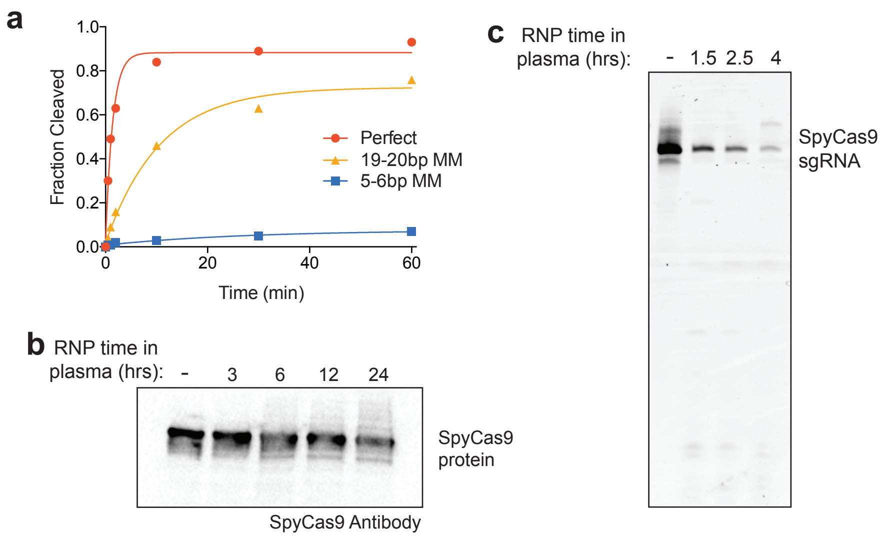

Supplementary Figure 3 | Mismatch cleavage by GeoCas9 and degradation of SpyCas9 in mammalian plasma

a, GeoCas9 cleavage of targets containing mismatches in the PAM proximal (5-6bp MM) or PAM distal (19-20bp MM) regions of the target DNA showing higher specificity for the PAM proximal region. **b,** Western blot against SpyCas9 after incubated the RNP in human plasma for the specified period of time. **c,** SpyCas9 sgRNA after incubation in human plasma for the specified period of time.

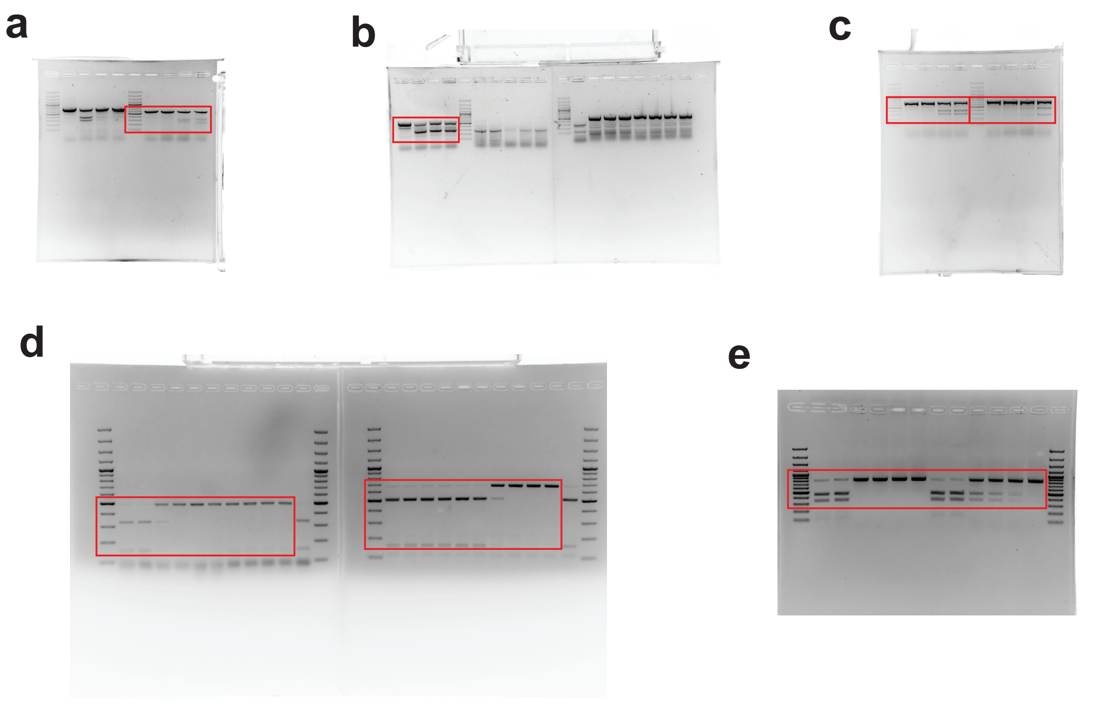

Supplementary Figure 4 | Uncropped images of gels for Figure 4 and 5

a, Uncropped gel shown in the top panel of Figure 4b. **b,** Uncropped gel shown in the bottom panel of Figure 4b. **c,** Uncropped gel shown in Figure 4c. **d,** Uncropped gel shown in the top panel of Figure 5a. **e,** Uncropped gel shown in Figure 5d. Red outline marks the region shown in the main text figures.
