# Supplementary material for: A thermostable Cas9 with increased lifetime in human plasma
Source: Nat Commun. 2017 Nov 10;8:1424. doi: 10.1038/s41467-017-01408-4 (PMC5681539; doi:10.1038/s41467-017-01408-4)
Supplement: Supplementary file 3 — Description of Additional Supplementary Files [file 41467_2017_1408_MOESM3_ESM.pdf]

Description of Additional Supplementary Files

File Name: Supplementary Data 1
Description: Thermophilic Cas9 candidates

File Name: Supplementary Data 2
Description: Spacers identified from G. st. and G. LC300

File Name: Supplementary Data 3
Description: DNA and RNA sequences used in this study
